# Case–Control Study of Toric Intraocular Lens Implantation in Congenital Cataract

**DOI:** 10.18502/jovr.v20.14282

**Published:** 2025-05-05

**Authors:** Lukpan Orazbekov, Neilya Aldasheva, Aidana Sutbayeva, Kairat Ruslanuly

**Affiliations:** ^1^First Ophthalmology Department, Kazakh Eye Research Institute, Almaty, Kazakhstan; ^2^Science Management Department, Kazakh Eye Research Institute, Almaty, Kazakhstan; ^3^Kazakhstan Medical University “Higher School of Public Health,” Almaty, Kazakhstan

**Keywords:** Congenital Cataract, Corneal Astigmatism, Intraocular Lens

## Abstract

**Purpose:**

To evaluate the visual and refractive outcomes after toric intraocular lens (IOL) implantation compared to monofocal IOLs in pediatric eyes with cataracts and preexisting corneal astigmatism.

**Methods:**

This case–control study was performed on 37 eyes of 37 children older than six years with visually significant congenital cataracts and corneal astigmatism greater than 1.75 diopters (D). The patients underwent lens aspiration with either monofocal (19 eyes) or toric (18 eyes) IOL implantation between June 2021 and December 2022. Uncorrected distance visual acuity (UCVA), corrected distance visual acuity (CDVA), near visual acuity, keratometry data, and residual astigmatism were evaluated preoperatively and on the fifth day as well as the first, third, and sixth months postoperatively.

**Results:**

Preoperative mean corneal astigmatism was 2.84 ± 0.51 D in the toric group and 3.05 ± 0.79 D in the non-toric group (*P* = 0.563). At the final follow-up, postoperative refractive astigmatism was 0.53 ± 0.33 D in the toric group and 2.33 ± 0.8 D in the non-toric group (*P* < 0.001). CDVA of 20/40 or better was achieved in 83.3% (*n* = 15) and 47.4% (*n* = 9) of eyes in the toric and non-toric groups, respectively (*P* = 0.038). Also, corrected near visual acuity of 20/40 or better was achieved in 100% (*n* = 18) and 78.9% (*n* = 15) of eyes in the toric and non-toric groups, respectively (*P* = 0.105).

**Conclusion:**

The study shows that pediatric cataract surgery with toric IOL implantation is an effective method of correcting preexisting corneal astigmatism. Compared to monofocal IOL implantation, it achieves better CDVA and near visual acuity.

##  INTRODUCTION

Currently, intraocular lens (IOL) implantation is widely used to treat congenital cataracts. Nevertheless, visual rehabilitation in patients with this condition remains challenging because of the unique problems, including residual astigmatism, associated with IOL implantation.^[1,2,3,4]^ Uncorrected astigmatism in early childhood can lead to meridional amblyopia, which may persist even after adequate optical correction.^[5,6,7,8]^

Correction of astigmatism with toric IOL may prevent the development of visual issues associated with residual astigmatism in children after pediatric cataract surgery (PCS). Although limited data exist regarding the effectiveness of toric IOL implantation in congenital cataracts,^[9,10,11]^ correcting corneal astigmatism with toric IOL implantation during cataract surgery is widely practiced and offers improved visual and refractive outcomes for adult patients.^[12,13,14,15]^ Toric IOL implantation in children is complicated by the challenge of accurately determining the degree of corneal astigmatism in a developing eye.

This study evaluates visual and refractive outcomes after toric IOL implantation in pediatric eyes with cataracts and preexisting corneal astigmatism.

##  METHODS

### Study Settings

This case–control study was performed on 37 eyes of 37 children who underwent lens aspiration using either monofocal (19 eyes) or toric (18 eyes) IOL implantation from June 2021 to December 2022. The study was conducted following the Declaration of Helsinki and was approved by the Local Ethics Committee at Kazakh Eye Research Institute, Kazakhstan (approval number №03-2021). The written informed consent was obtained from all parents.

### Eligibility Criteria 

Inclusion criteria:(1) visually significant congenital cataract; (2) corneal astigmatism greater than 1.75 diopters (D); and (3) age greater than six years.

Exclusion criteria:(1) patients with corneal diameter < 9 mm; (2) corneal scarring; (3) irregular astigmatism; (4) intraocular pressure > 25 mmHg; (5) active uveitis or signs of a previous episode of uveitis; (6) traumatic, subluxated, or complicated cataract; (7) persistent hyperplastic primary vitreous (anterior or posterior); (8) optic nerve or macular disease that might limit visual potential; and (9) history of previous intraocular surgery.

### Preoperative and Postoperative Evaluations

Each patient underwent a complete ophthalmic examination before the surgery, during the follow-up period on the fifth day, and at the first, third, and sixth months postoperatively. This included a slit-lamp examination, assessment of uncorrected distance visual acuity (UCVA), corrected distance visual acuity (CDVA), near visual acuity, dilated fundus examination with indirect ophthalmoscopy, keratometry, and refraction measurements.

For better numerical representations and convenience of analysis, we used logMAR units to express distance and near visual acuity. UCVA and CDVA were measured using the Snellen chart, and near visual acuity was tested within the range of 20 to 40 cm using the Radner Reading Chart and converted to logMAR for analysis.

Ocular biometry was measured using ZEISS IOLMaster 700, a swept-source optical coherence tomography-based biometer (Carl Zeiss Meditec AG). If axial length could not be obtained using this biometer, an immersion ultrasound A-scan was performed using the Ocuscan system (Alcon Laboratories Inc.).

Keratometry was performed using Pentacam (Oculus), and postoperative refractive error was obtained with a KR-1W Wavefront Analyzer (Topcon).

The IOL power was calculated using the Barrett Universal II formula with the Measurement Module of the Verion Image Guided System (Alcon Laboratories Inc). IOLs were implanted based on the calculated target refraction, and any under-correction was avoided. In the non-toric group, the target refraction was set to 0 D in spherical equivalent. The folding hydrophobic acrylic IOL named AcrySof IQ SN60WF (Alcon Laboratories Inc) was implanted for the non-toric group, and AcrySof IQ Toric SN6AT (Alcon Laboratories Inc) was chosen for the toric group.

### Surgical Technique

One experienced surgeon (LO) performed all procedures under general anesthesia using the standard technique of PCS with IOL implantation. In the case of toric IOL implantation, the Verion Digital Marker (Alcon Laboratories Inc) overlay was used for IOL alignment. Paracentesis was performed in both upper quadrants, and a high-molecular-weight ophthalmic viscosurgical device (OVD) was injected into the anterior chamber. Then, a 2.4 mm corneal tunnel incision was made at 11 o'clock with a disposable keratome knife (2.2 mm). Circular continuous capsulorhexis was approximately 5.5 mm. Сortical cleaving hydrodissection was followed by bimanual phacoaspiration in irrigation-aspiration mode. The OVD was injected into the anterior chamber, and an IOL was implanted in the capsular bag. Residual OVD was aspirated from the anterior chamber and behind the IOL. Next, the IOL was carefully aligned, and the surgical procedure was completed with corneal hydration.

All patients received combined antibacterial and anti-inflammatory eye drops for one month after surgery.

### Statistical Analysis

An independent statistician performed statistical analysis using GraphPad Prism 8 (GraphPad Software Inc.). All data were expressed in means ± standard deviation and range. Shapiro-Wilk test was conducted to evaluate whether datasets were normally distributed. Nonparametric tests, including the Wilcoxon signed-rank test and the Mann–Whitney U test, were used to analyze continuous data. Fisher's Exact Test was applied for categorical data to assess the associations. The nonparametric Friedman test was used as an alternative to one-way ANOVA for one-way repeated measures analysis of variance by ranks. Post-hoc pairwise comparisons were performed using the Wilcoxon signed-rank test with Bonferroni correction to identify significant differences among specific follow-up periods. In all analyses, *P* < 0.05 was considered statistically significant.

##  RESULTS

A total of 37 eyes were included in this study: 18 eyes received toric IOLs and 19 eyes underwent non-toric IOL implantation. The patients' demographic characteristics are shown in Table 1. Both groups were comparable in terms of all preoperative clinical parameters except for the mean IOL power in spherical diopters (*P* = 0.004). No loss to follow-up or missed appointments was observed during this study.

### Visual Acuity

**Figure 1:**
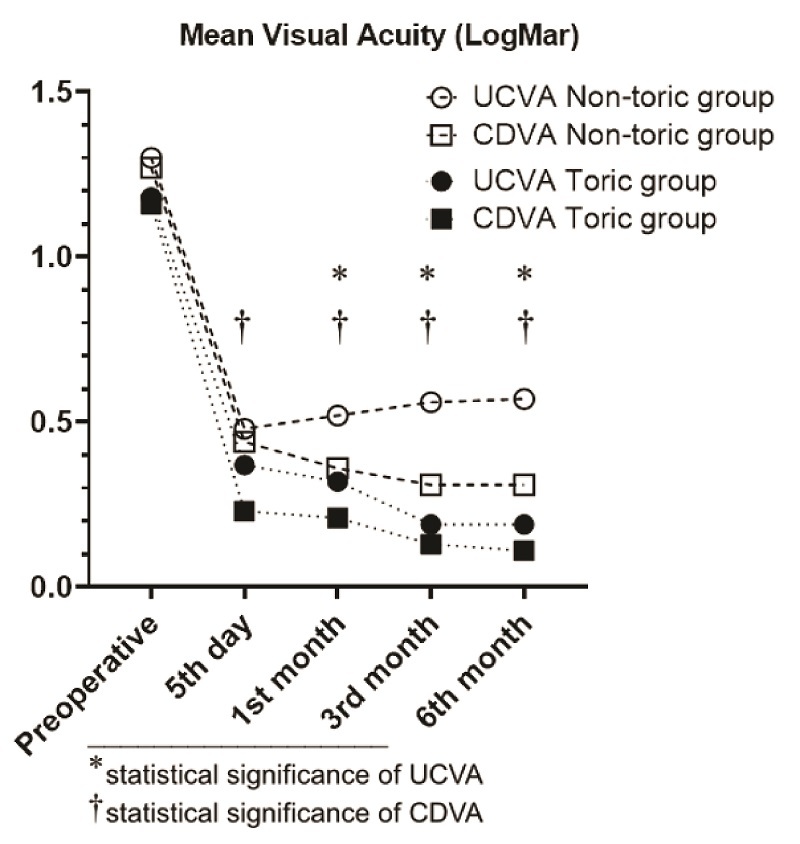
The meanpreoperative and postoperative uncorrected (UCVA) and corrected (CDVA) distance visual acuity in the toric and non-toric groups in terms of logMAR units.

Preoperative and postoperative UCVA and CDVA of patients are presented in Table 2. There was no statistically significant difference between preoperative UCVA and CDVA of the toric and non-toric groups (*P *= 0.89 and *P *= 0.81, respectively). Improvement of UCVA and CDVA of toric and non-toric groups showed no statistically significant difference between the two groups (*P* = 0.529 and *P* = 0.568, respectively). The preoperative and postoperative UCVA and CDVA curves are shown in Figure 1.

**Figure 2:**
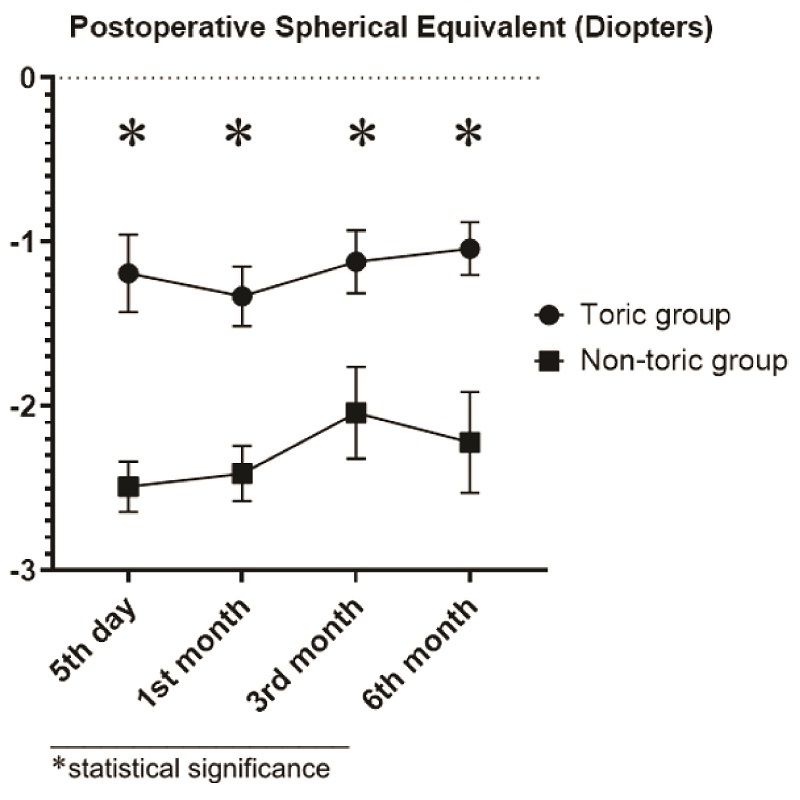
Postoperative spherical curve (in diopters) in the toric and non-toric groups, represented in the mean and standard error of the mean.

**Figure 3:**
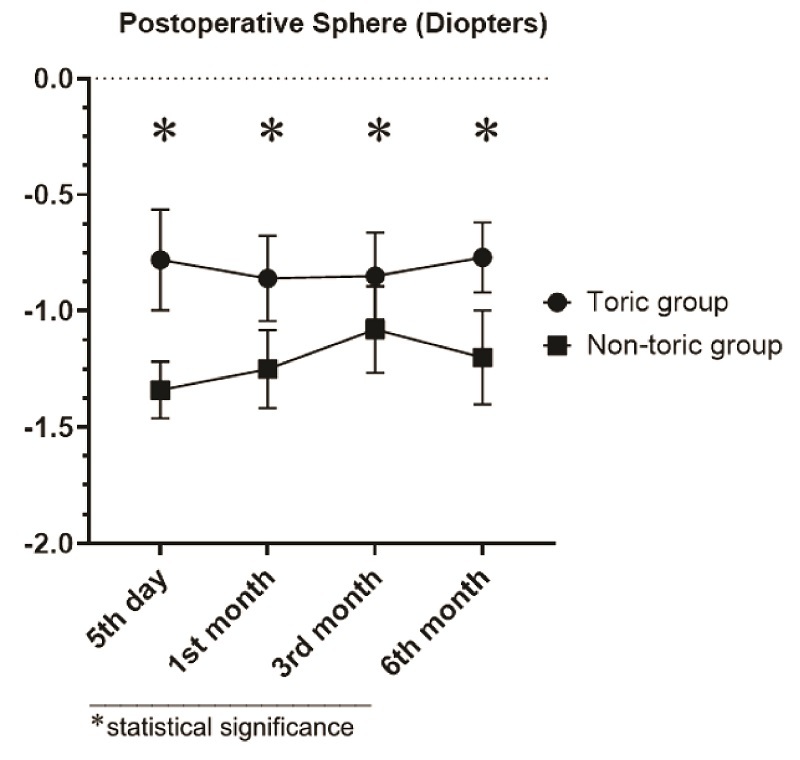
Postoperative spherical equivalent curve (in diopters) in the toric and non-toric groups, represented in the mean and standard error of the mean.

At the final follow-up, CDVA of 20/25 or better was achieved in 66.7% (*n* = 12) and 0% (*n* = 0) of eyes in the toric and non-toric groups, respectively *(P* < 0.001). Also, CDVA of 20/40 or better was achieved in 100% (*n* = 18) and 47.4% (*n* = 9) of eyes in the toric and non-toric groups, respectively (*P* < 0.001). Uncorrected and corrected near visual acuity of 20/40 or better was achieved in 66.7% (*n* = 12) and 100% (*n* = 18) of eyes in the toric group, respectively (*P* = 0.191). On the other hand, uncorrected and corrected near visual acuity of 20/40 or better was achieved in 42.1% (*n* = 8) and 78.9% (*n* = 15) of eyes in the non-toric group, respectively (*P* = 0.045). Postoperative sphere and spherical equivalent curves are shown in Figures 2 and 3.

### Toric Group 

We observed a steady decrease in logMAR UCVA and CDVA of 0.98 ± 0.55 and 1.05 ± 0.51, respectively, during six months of follow-up. On the fifth day after surgery, there was a decrease in logMAR UCVA and CDVA of 0.81 ± 0.50 and 0.93 ± 0.54, respectively. From the fifth day to the first month of follow-up, there was a decrease in logMAR UCVA and CDVA of 0.05 ± 0.15 and 0.02 ± 0.11, respectively. From the first month to the third month of follow-up, there was a decrease in logMAR UCVA and CDVA of 0.13 ± 0.17 and 0.08 ± 0.15, respectively. From the third month to the sixth month of follow-up, logMAR UCVA remained stable but logMAR CDVA slightly decreased (0.02 ± 0.03 logMAR).

**Table 1:** Demographic characteristics of patients with pediatric cataract and corneal astigmatism

**Characteristics**	**Group toric**	**Group non-toric**	***P*** **-value** ${\;}^{†}$
Eyes (*n*)	18	19	NA
Mean age ± SD (range) years	9.05 ± 2.19 (6 to 12)	9.05 ± 1.79 (6 to 12)	0.922
Mean IOL power in spherical diopter (range)	25.21 ± 2.67 D (20.50 to 29.50 D)	22.64 ± 2.09 D (20.00 to 27.00 D)	0.004 ${\;}^{*}$
Mean IOL power in cylindrical diopter (range)	3.01 ± 0.52 D (2.25 to 4.00 D)	–	NA
Axial length in mm (range)	22.21 ± 0.89 (21.01–23.93)	22.64 ± 2.09 (20.0–26.5)	0.946
SD, standard deviation; IOL, intraocular lens; D, diopters; NA, not applicable			
${\;}^{*}$ Statistically significant			
${\;}^{†}$ Nonparametric Mann–Whitney U test is used to analyze data between two independent groups			

**Table 2:** Visual acuity before pediatric cataract surgery and during the follow-up period (day five, month one, month three, and month six)

**Visual acuity (LogMAR)**	**Mean ± SD; Range**					***P*** **-value** ${\;}^{‡}$
	**Preoperative**	**Postoperative**				
		**Fifth day**	**First month**	**Third month**	**Sixth month**	
Toric group's UCVA	1.18 ± 0.52; 0.52 to 2.3	0.37 ± 0.2; 0.15 to 0.69	0.32 ± 0.19; 0.15 to 0.69	0.19 ± 0.13; 0.09 to 0.53	0.19 ± 0.13; 0.09 to 0.53	< 0.001 ${\;}^{*}$
Non-toric group's UCVA	1.3 ± 0.39; 0.69 to 2.3	0.48 ± 0.2; 0.22 to 1.0	0.52 ± 0.2; 0.22 to 1.0	0.56 ± 0.21; 0.3 to 1.0	0.57 ± 0.21; 0.3 to 1.0	< 0.001 ${\;}^{*}$
*P*-value ${\;}^{†}$ for UCVA	0.529	0.097	0.003 ${\;}^{*}$	< 0.001 ${\;}^{*}$	< 0.001 ${\;}^{*}$	
Toric group's CDVA	1.16 ± 0.51; 0.52 to 2.3	0.23 ± 0.17; 0.09 to 0.69	0.21 ± 0.15; 0.09 to 0.69	0.13 ± 0.04; 0.09 to 0.22	0.11 ± 0.04; 0.09 to 0.22	< 0.001 ${\;}^{*}$
Non-toric group's CDVA	1.27 ± 0.44; 0.52 to 2.3	0.44 ± 0.25; 0.15 to 1.0	0.36 ± 0.25; 0.15 to 1.0	0.31 ± 0.17; 0.15 to 0.69	0.31 ± 0.17; 0.15 to 0.52	< 0.001 ${\;}^{*}$
*P*-value ${\;}^{†}$ for CDVA	0.568	0.006 ${\;}^{*}$	0.012 ${\;}^{*}$	< 0.001 ${\;}^{*}$	< 0.001 ${\;}^{*}$	
SD, standard deviation; UCVA, uncorrected visual acuity; CDVA, corrected distance visual acuity; LogMAR, Logarithm of the Minimum Angle of Resolution						
${\;}^{*}$ Statistically significant						
${\;}^{†}$ Nonparametric Mann–Whitney U test is used to analyze data between two independent groups						
${\;}^{‡}$ Nonparametric Friedman test is used to analyze repeated measures data						

### Non-toric (Control) Group

We observed a decrease in logMAR UCVA and CDVA of 0.71 ± 0.42 and 0.91 ± 0.49, respectively, over the course of the six-month follow-up. On the fifth day after surgery, there was a decrease in logMAR UCVA and CDVA of 0.82 ± 0.36 and 0.83 ± 0.45, respectively. From the fifth day to the first month of follow-up, there was an increase in logMAR UCVA and a decrease in logMAR CDVA of 0.03 ± 0.08 and 0.08 ± 0.22, respectively. From the first month to the third month of follow-up, there was an increase in logMAR UCVA and a decrease in logMAR CDVA of 0.05 ± 0.12 and 0.05 ± 0.17 logMAR, respectively. Finally, between the third month and the sixth month of follow-up, there was an increase in logMAR UCVA and CDVA of 0.03 ± 0.07 and 0.04 ± 0.07, respectively.

### Post-hoc Statistical Analysis of Visual Acuity

A post-hoc analysis was performed to further assess the changes in visual acuity across the follow-up periods. For UCVA, the Friedman test indicated significant differences across time points for both the toric ( χ 2 = 25.78, *P* < 0.001) and control groups ( χ 2 = 23.62, *P* < 0.001). Post-hoc analysis revealed significant improvements in UCVA from the preoperative to the fifth day (*P* < 0.05, Bonferroni corrected) for both groups. No significant differences were observed between the one-month, three-month, and six-month follow-ups, indicating stability after the initial improvement. Similar trends were observed in terms of CDVA, with the Friedman test showing significant differences for both the toric ( χ 2 = 22.43, *P* < 0.001) and control ( χ 2 = 21.08, *P* < 0.001) groups. Significant gains in CDVA were seen from the preoperative to the fifth day (*P* < 0.05, Bonferroni corrected). There were no significant changes between the follow-ups at one month, three months, and six months, indicating stable visual outcomes. These findings demonstrate that the most substantial improvements in UCVA and CDVA occurred early, by the fifth day postoperatively, and were maintained throughout the six-month follow-up period.

### Refractive Astigmatism

There was a statistically significant difference in the residual refractive cylinder between the two groups at day five, month one, month three, and month six postoperatively (*P* < 0.001 for all time points) [Table 3].

A residual refractive cylinder of 0.50 D or less was observed in 61.1% (*n* = 11) of eyes in the toric group and 0% (*n* = 0) in the non-toric groups (*P* < 0.001). Additionally, a residual refractive cylinder of 1.00 D or less was observed in 94.4% (*n* = 17) of eyes in the toric group and 5.3% (*n* = 1) in the non-toric group (*P* < 0.001). At the final six-month follow-up, postoperative refractive astigmatism was 0.53 ± 0.33 D in the toric group and 2.33 ± 0.8 D in the non-toric group (*P* < 0.001).

**Table 3:** Postoperative refractive error (day five, month one, month three, and month six)

**Parameter**		**Mean ± SD; Range**				***P*** **-value** ${\;}^{†}$
		**Postoperative**				
		**Fifth day**	**First month**	**Third month**	**Sixth month**	
Toric group	Sphere (D)	–0.78 ± 0.92; –3.27 to 1.25	–0.86 ± 0.78; –3.15 to 0.52	–0.85 ± 0.79; –3.15 to 0.52	–0.77 ± 0.64; –2.15 to 0.52	0.659
	Cylinder (D)	–0.84 ± 0.72; –1.75 to 1.0	–0.94 ± 0.59; –1.8 to 0.52	–0.54 ± 0.35; –1.25 to –0.15	–0.53 ± 0.33; –1.25 to –0.15	0.029 ${\;}^{*}$
	Refractive cylinder (D)	0.96 ± 0.53; 0.1 to 1.75	1.01 ± 0.45; 0.1 to 1.8	0.54 ± 0.34; 0.15 to 1.25	0.53 ± 0.33; 0.15 to 1.25	0.002 ${\;}^{*}$
Control group	Sphere (D)	–1.34 ± 0.53; –2.25 to –0.5	–1.25 ± 0.73; –2.25 to 0.75.	–1.08 ± 0.81; –2.25 to 1.25	–1.2 ± 0.88; –2.25 to 1.25	0.693
	Cylinder (D)	–2.31 ± 0.69; –3.75 to –1.25	–2.33 ± 0.68; –3.75 to –1.5	–1.92 ± 1.32; –3.75 to 2.25	–2.04 ± 1.41; –3.75 to 2.75	0.301
	Refractive cylinder (D)	2.31 ± 0.69; 1.25 to 3.75	2.33 ± 0.68; 1.5 to 3.75	2.16 ± 0.85; 1.0 to 3.75	2.33 ± 0.8; 1.0 to 3.75	0.884
SD, standard deviation; SE, spherical equivalent; D, diopters						
${\;}^{*}$ Statistically significant						
${\;}^{†}$ Nonparametric Wilcoxon signed-rank test is used to analyze the data from the fifth day and sixth month postoperative periods of the same group						

**Table 4:** Corneal astigmatism and keratometry before pediatric cataract surgery and during the follow-up period (day five, month one, month three, and month six)

**Parameter**		**Mean ± SD; Range**					***P*** **-value** ${\;}^{‡}$
		**Preoperative**	**Postoperative**				
			**Fifth day**	**First month**	**Third month**	**Sixth month**	
Toric group	Flat K, D	41.84 ± 0.98; 40.16 to 44.0	42.05 ± 0.96; 40.5 to 44.0	41.99 ± 0.77; 40.75 to 43.77	41.72 ± 0.64; 40.2 to 42.77	41.69 ± 0.98; 40.12 to 43.1	0.064
	Steep K, D	44.69 ± 0.94; 43.2 to 46.25	44.82 ± 0.93; 43.5 to 46.25	44.79 ± 0.73; 43.5 to 46.0	44.49 ± 0.76; 43.0 to 45.5	44.5 ± 1.09; 42.9 to 46.25	< 0.001 ${\;}^{*}$
	Km, D	43.27 ± 0.92; 41.69 to 45.13	43.5 ± 0.83; 42.0 to 45.13	43.39 ± 0.74; 41.98 to 44.81	43.11 ± 0.68; 41.6 to 44.0	43.09 ± 1.02; 41.66 to 44.63	0.432
	CA, D	2.84 ± 0.51; 2.04 to 3.95	2.76 ± 0.53; 2.0 to 3.95	2.8 ± 0.45; 2.0 to 3.74	2.77 ± 0.76; 2.2 to 3.25	2.81 ± 0.42; 2.12 to 3.46	< 0.001 ${\;}^{*}$
Non-toric group	Flat K, D	41.24 ± 0.76; 40.1 to 42.5	41.28 ± 0.76; 40.0 to 42.5	41.28 ± 0.79; 40.2 to 42.6	41.29 ± 0.71; 40.1 to 42.4	41.02 ± 0.63; 40.1 to 42.16	0.115
	Steep K, D	44.28 ± 0.79; 42.75 to 46.2	44.21 ± 0.73; 42.75 to 45.6	44.14 ± 0.66; 42.75 to 45.45	44.12 ± 0.66; 42.75 to 45.45	43.92 ± 0.66; 42.43 to 45.4	0.051
	Km, D	42.76 ± 0.67; 41.72 to 44.35	42.75 ± 0.65; 41.72 to 44.05	42.71 ± 0.61; 41.72 to 43.81	42.71 ± 0.59; 41.72 to 43.85	42.47 ± 0.59; 41.27 to 43.78	0.031 ${\;}^{*}$
	CA, D	3.05 ± 0.79; 2.05 to 4.5	2.93 ± 0.74; 2.05 to 4.7	2.85 ± 0.8; 2.0 to 4.6	2.83 ± 0.68; 2.05 to 4.5	2.89 ± 0.47; 2.2 to 3.9	0.646
*P*-value ${\;}^{†}$ for CA		0.563	0.542	0.668	0.792	0.769	
SD, standard deviation; K, keratometry; Km, mean keratometry; CA, corneal astigmatism; D, diopters							
${\;}^{*}$ Statistically significant							
${\;}^{†}$ Nonparametric Mann–Whitney U test is used to analyze outcomes between two independent groups							
${\;}^{‡}$ Nonparametric Friedman test is used to analyze repeated measures data							

### Corneal Astigmatism

Comparing the preoperative and postoperative keratometry parameters revealed no statistically significant difference between the two study groups (*P* > 0.05) [Table 4]. All patients in the two groups had a preoperative corneal astigmatism of 2.00 D and more, and the prevalence of preoperative corneal astigmatism of 3.00 D or more was 44.4% and 36.8% in the toric and non-toric groups, respectively (*P* = 0.743). In the toric group, nine eyes had against-the-rule astigmatism, eight had with-the-rule astigmatism, and one had oblique astigmatism. In the non-toric group, eleven eyes had against-the-rule astigmatism, seven had with-the-rule astigmatism, and one had oblique astigmatism.

Preoperative corneal astigmatism was 2.84 ± 0.51 D in the toric group and 3.05 ± 0.79 D in the non-toric group (*P* = 0.563). At the final follow-up at six months, corneal astigmatism was 2.81 ± 0.42 and 2.89 ± 0.47 in the toric and non-toric groups, respectively (*P *= 0.769). When comparing the two groups at each time point, no statistically significant differences were observed (*P* > 0.05). However, the repeated measures analysis showed a significant reduction in corneal astigmatism within the toric group (*P* < 0.001), whereas no significant change was observed in the non-toric group (*P* = 0.646).

A comparison of preoperative corneal astigmatism and postoperative refractive astigmatism showed a statistically significant difference in both groups (*P* < 0.001 and *P*= 0.004 in toric and non-toric groups, respectively).

### Complications

There were neither intraoperative nor postoperative complications in either group in the six-month follow-up period.

##  DISCUSSION

Emmetropization commonly occurs at age six, when refractive anomalies, astigmatism, and anisometropia are minimized in most children.^[16,17]^ Therefore, this study included children older than six to exclude any potential influence of myopic shift, which could otherwise impact the results.

Woodruff studied 631 children between the ages of one and six, and the findings showed that astigmatism tends to increase with age.^[18]^ However, Gwiazda et al studied 1000 children less than six years of age and reported opposite results.^[19]^ They observed that both the incidence and magnitude of astigmatism are highest in the first two years, after which they decline. Furthermore, children with no astigmatism in the first year of life rarely acquire it later. Those with significant astigmatism in the first year either have it eliminated or significantly reduced over the years. Based on the findings of both these studies, astigmatism is against the rule in the developing globe of younger children. They suggest that with age, most of the against-the-rule astigmatism is either gradually eliminated or changes to with-the-rule, possibly because of the increasing pressure by the eyelids over time.

Regarding the association of astigmatism with congenital cataracts, it appears that anterior segment anomalies often complicate congenital cataracts due to their close anatomical location and embryonic origin.^[20,21]^ Higher values of corneal astigmatism and central corneal thickness are correlated with the location of lens opacities. Accordingly, the highest corneal astigmatism and central corneal thickness were found in patients with anterior cataracts, and as lens opacities became more posterior, corneal astigmatism and central corneal thickness gradually decreased from 2.50 D to 0.30 D and from 623 mm to 555 mm, respectively. Eyes with total and anterior cataracts had a smaller anterior chamber depth of approximately 2.80 mm, whereas eyes with posterior cataracts had a larger anterior chamber depth of up to 4.1 mm.

Previously, Orazbekov et al conducted a case–control study on monofocal IOL implantation during congenital cataract surgery in children aged 4 to 80 months and observed that 78.32% of these children exhibited corneal astigmatism > 1.00 D; this level of astigmatism persisted in 89.29% of these children at seven years of age.^[2]^ According to other studies, the worldwide prevalence of corneal astigmatism > 1.00 D in congenital cataracts ranges from 59.71% to 79.0%, with the mean keratometry declining from 51.2 D in newborns to 43.5 D in older children.^[22,23,24]^ Thus, we questioned the rationale for toric IOL implantation in children with congenital cataracts based on keratometry data. A literature review showed limited data regarding the use of toric IOLs in children, and the existing studies either had a retrospective design or lacked a control group for comparison.^[9,10,11]^

In a prospective randomized study, Ram et al compared visual and refractive outcomes after PCS with toric and non-toric IOL implantation. Their analysis was based on 21 eyes of 17 children (8–14 years) with congenital cataracts and corneal astigmatism > 1.50 D.^[9]^ The mean preoperative corneal astigmatism was 2.99 ± 0.96 D (range, 1.85–5.12 D) in the toric group and 3.35 ± 0.63 D (range, 2.03–4.33 D) in the non-toric group (*P* = 0.31). Mean postoperative refractive astigmatism at one-year follow-up was 0.50 ± 0.39 D (range, 0.00–1.00 D) in the toric group and 2.05 ± 0.39 D (range, 1.25–2.50 D) in the non-toric group (*P* = 0.006). In the toric group, preoperative UCVA and CDVA were 0.94 ± 0.51 and 0.72 ± 0.17 logMAR, which improved postoperatively to 0.51 ± 0.33 and 0.30 ± 0.21 logMAR, respectively. In the control group, preoperative UCVA and CDVA were 1.52 ± 1.12 and 1.33 ± 1.08 logMAR, which improved postoperatively to 0.77 ± 0.68 and 0.30 ± 0.21 logMAR, respectively. At the final follow-up, CDVA of 20/30 or better was achieved in 36.4% and 10.0% of eyes in the toric and non-toric groups, respectively.

In their retrospective interventional case study, Tachibana et al compared 36 eyes of 26 patients (aged 3–16 years) with congenital cataracts and corneal astigmatism > 1.50 D.^[10]^ The patients underwent PCS using either toric IOLs with optic capture or non-toric IOLs. In the present study, the mean preoperative corneal astigmatism was 3.30 ± 1.20 D (range, 1.30–4.80 D) in the toric group and 2.50 ± 1.10 D (range, 1.00–5.10 D) in the non-toric group (*P* = 0.081). The mean one-year postoperative refractive astigmatism was 1.38 ± 0.80 D (range, 0.40–3.50 D) in the toric group, significantly smaller than the mean corneal astigmatism (*P* = 0.0001). The mean preoperative CDVA was 0.57 logMAR in the toric group and 0.71 logMAR in the non-toric group, with logMAR decreasing to 0.003 and 0.09, respectively, one year postoperatively. No statistically significant difference was observed regarding the mean preoperative (*P* = 0.435) or postoperative (*P* = 0.464) CDVA between the two groups.

In a prospective interventional case study, Vasavada et al performed PCS with toric IOL implantation in 76 eyes of 51 children (5–16 years) with congenital cataract and corneal astigmatism > 1.50 D.^[11]^ The authors did not report on the mean preoperative UCVA; however, the mean postoperative UCVA was 0.32 ± 0.26 logMAR. CDVA improved from 0.59 ± 0.43 preoperatively to 0.23 ± 0.27 logMAR postoperatively at the third-year follow-up (*P* = 0.03). The mean preoperative corneal astigmatism was 1.56 ± 2.13 D. At the third-year follow-up, the mean refractive astigmatism was 0.55 ± 0.40 D. Furthermore, UCVA and CDVA of 20/40 or better were achieved in 73.68% and 81.57% of eyes, respectively.

In all of the studies above involving toric IOL implantation, some children showed poor UCVA and CDVA, which could possibly be attributed to factors such as anisometropia, deprivation amblyopia, or meridional amblyopia. In the toric group of our study, preoperative UCVA and CDVA were 1.18 ± 0.52 and 1.16 ± 0.51 logMAR, which significantly improved to 0.19 ± 0.13 and 0.11 ± 0.04 logMAR after surgery, respectively. In the toric group, the mean preoperative corneal astigmatism was 2.84 ± 0.51 D, and the mean postoperative refractive astigmatism was 0.53 ± 0.33 D. In the non-toric group, the mean corneal astigmatism was 3.05 ± 0.79 D before surgery, and the mean refractive astigmatism was 2.33 ± 0.8 D at one-year follow-up which is significantly higher compared to the toric group (*P* < 0.001). At the final follow-up, CDVA of 20/25 or better was achieved in 66.7% and 0% of eyes in the toric and non-toric groups, respectively. In addition, a CDVA of 20/40 or better was achieved in 83.3% and 47.4% of eyes in the toric and non-toric groups, respectively. Uncorrected and corrected near VA of 20/40 or better were achieved in 66.7% and 100% of eyes in the toric group, respectively, and 42.1% and 78.9% in the non-toric group, respectively.

In our study, there was a 2.5 D difference in the mean IOL power between the two groups, which may be attributed to several ocular characteristics that differed between the two groups. Thus, compared to the toric group, the non-toric group exhibited a higher axial length (with a mean difference of approximately 0.43 mm) and a flatter cornea (with a mean keratometry difference of approximately 0.51 D). The present study had no posterior capsule opacification, glaucoma, or postoperative inflammatory reaction cases. Other studies focusing on toric IOL implantation in children have reported posterior capsule opacification in up to 5.3% of cases; however, similar to our research, there have been no cases of glaucoma or postoperative inflammation. ${\;}^{\left[9--11\right]}$

The main limitations of our study are the relatively small sample size and the short-term follow-up after toric IOL implantation in children. In summary, the results of our study demonstrated that cataract surgery with toric IOL implantation, compared to monofocal IOL implantation, yields favorable results in correcting preexisting corneal astigmatism and improving UCVA and CDVA in children.

##  Financial Support and Sponsorship

None.

##  Conflicts of Interest

None.
